# Strategies for Effective Use of Genomic Information in Crop Breeding Programs Serving Africa and South Asia

**DOI:** 10.3389/fpls.2020.00353

**Published:** 2020-03-27

**Authors:** Nicholas Santantonio, Sikiru Adeniyi Atanda, Yoseph Beyene, Rajeev K. Varshney, Michael Olsen, Elizabeth Jones, Manish Roorkiwal, Manje Gowda, Chellapilla Bharadwaj, Pooran M. Gaur, Xuecai Zhang, Kate Dreher, Claudio Ayala-Hernández, Jose Crossa, Paulino Pérez-Rodríguez, Abhishek Rathore, Star Yanxin Gao, Susan McCouch, Kelly R. Robbins

**Affiliations:** ^1^Section of Plant Breeding and Genetics, School of Integrative Plant Sciences, Cornell University, Ithaca, NY, United States; ^2^West Africa Center for Crop Improvement (WACCI), University of Ghana, Accra, Ghana; ^3^International Maize and Wheat Improvement Center (CIMMYT), Texcoco, Mexico; ^4^International Maize and Wheat Improvement Center (CIMMYT), Nairobi, Kenya; ^5^Center of Excellence in Genomics & Systems Biology, International Crops Research Institute for the Semi-Arid Tropics (ICRISAT), Hyderabad, India; ^6^Genomic Open-Source Breeding Informatics Initiative (GOBii) Project, Institute of Biotechnology, Cornell University, Ithaca, NY, United States; ^7^Division of Genetics, Indian Agriculture Research Institute (ICAR), New Delhi, India; ^8^Research Program - Asia, International Crops Research Institute for the Semi-Arid Tropics (ICRISAT), Hyderabad, India; ^9^Colegio de Postgraduados, Mexico, Mexico

**Keywords:** genomic selection, genomic prediction, breeding informatics, breeding scheme optimization, plant breeding, trial design

## Abstract

Much of the world’s population growth will occur in regions where food insecurity is prevalent, with large increases in food demand projected in regions of Africa and South Asia. While improving food security in these regions will require a multi-faceted approach, improved performance of crop varieties in these regions will play a critical role. Current rates of genetic gain in breeding programs serving Africa and South Asia fall below rates achieved in other regions of the world. Given resource constraints, increased genetic gain in these regions cannot be achieved by simply expanding the size of breeding programs. New approaches to breeding are required. The Genomic Open-source Breeding informatics initiative (GOBii) and Excellence in Breeding Platform (EiB) are working with public sector breeding programs to build capacity, develop breeding strategies, and build breeding informatics capabilities to enable routine use of new technologies that can improve the efficiency of breeding programs and increase genetic gains. Simulations evaluating breeding strategies indicate cost-effective implementations of genomic selection (GS) are feasible using relatively small training sets, and proof-of-concept implementations have been validated in the International Maize and Wheat Improvement Center (CIMMYT) maize breeding program. Progress on GOBii, EiB, and implementation of GS in CIMMYT and International Crops Research Institute for the Semi-Arid Tropics (ICRISAT) breeding programs are discussed, as well as strategies for routine implementation of GS in breeding programs serving Africa and South Asia.

## Introduction

Crop improvement through plant breeding is a process of continuous genetic improvement through selection and recombination of superior lines. The response to selection, or rate of genetic gain, is dependent on multiple factors, expressed in the “breeder’s equation,”

(1) $$R=\frac{i\,r\,\sigma_{a}}{L}$$

where *R* is the response to selection per year, *i* is the selection intensity, *r* is the accuracy of selection, **σ**_*a*_ is the additive genetic standard deviation for the trait of interest, and *L* is the generation interval (Lush, 1936).

Assuming that breeding objectives, selection criteria, available germplasm, and target environments are well defined, the success of a breeding program is largely dependent on the optimal use of available resources to maximize the response to selection (Rutkoski, 2019). Effective breeding programs must re-evaluate breeding strategies as technology, environments, access to germplasm, and consumer needs are constantly changing. While all the aforementioned factors are critical, the ability to identify and effectively implement new technologies can be challenging. This is especially true for publicly funded breeding programs in Africa and South Asia, where resource and infrastructure limitations make the adoption of new technologies particularly challenging. The need to overcome these limitations and improve the effectiveness of breeding programs is urgent, given the historically low rates of genetic gains in many programs serving Africa and South Asia (Godfray et al., 2010; Cobb et al., 2019), expected population growth (Alexandratos and Bruinsma, 2012), and the potential impacts of climate change on crop production (Ritchie et al., 2018).

To achieve rates of genetic gain in crop improvement needed to strengthen and stabilize food security, modern technologies must be adopted and efficiently implemented. One promising approach is genomic selection (GS), where the performance of new lines is predicted based on genome-wide information (Meuwissen et al., 2001). Multiple studies have shown the potential of this methodology to increase the rates of genetic gain in plant breeding programs (Heffner et al., 2009; Beyene et al., 2015; Gaynor et al., 2017; Crossa et al., 2017; Rutkoski et al., 2017), often through the reduction in cycle time, *L*. However, despite compelling evidence of the potential gains from GS and widespread adoption in animal breeding, public sector plant breeding programs have been slow to routinely adopt GS. Adoption of GS in animal breeding applications benefited from the fact that the use of genomic Best Linear Unbiased Predictors (GBLUP) (VanRaden, 2008) and single-step GBLUP (Legarra et al., 2014) enabled GS implementations that were straightforward extensions of existing breeding approaches. In contrast, optimal implementations of GS in plant breeding programs represent a significant change in how breeding data is analyzed, how breeding decisions are made, and how breeding pipelines are designed. The costs and challenges of large-scale implementation of genomic selection in public sector breeding programs have been a significant barrier to routine implementation despite the potential for significant increases in genetic gain.

A typical inbred or hybrid plant breeding program has this basic structure: (i) selection of parents for crossing, (ii) selfing or use of doubled haploid technology (DH) to achieve the desired level of homozygosity, and (iii) multi-stage field trials of selection candidates (inbred lines or testcross hybrids) to identify best lines or hybrids for release and commercialization as varieties. We generalize this structure as a variety development pipeline (VDP, e.g., Figure 4 of Cooper et al., 2014). A typical VDP evaluates progeny lines in the field for several growing seasons, advancing the best lines at the end of each season, with smaller numbers of lines being tested in more environments in each successive season. Lines that are deemed successful in advanced trials are candidates for variety release and are typically recycled as parents into the breeding program. This approach to breeding takes advantage of the ability to produce inbred or testcross hybrid seed in large quantities which is then extensively evaluated in the field. In this approach, decisions to recycle lines as new parents are made using extensive, but costly, phenotypic data, often with lines treated as independent factor levels in the analysis. This approach produces accurate (*r*, Equation 1) estimates of line performance but significantly increases generation interval (*L*, Equation 1) due to the multiple years of testing. While simulation studies demonstrate that a rapid-cycle recurrent GS approach may ultimately provide the largest increases in genetic gains (Gaynor et al., 2017; Gorjanc et al., 2018; Rembe et al., 2019), it is not a practical initial implementation of GS in a plant breeding program. Rapid cycle approaches require relatively large training sets that must be routinely updated to maintain prediction accuracy and breeding decisions must be made using less accurate estimates of the line performance, often without observing the line in replicated trials (Crossa et al., 2010; Hickey et al., 2014; Schopp et al., 2017; Gorjanc et al., 2018). This represents a significant change in how breeding decisions are made and requires significant investments for training set development. Both of these factors can limit adoption of GS, especially in resource limited breeding programs, and these factors need to be considered when developing a strategy for implementation of GS.

Large scale adoption of GS will require optimizing breeding strategies while accounting for costs, ease of implementation, and potential impacts on operation efficiency and genetic gain. Ideally, training data for a rapid-cycle recurrent selection approach would be sourced from the breeding program’s VDP. So, regardless of the ultimate end goal and long-term GS strategy, the first step in GS implementation is to routinely genotype lines entering the VDP. For sustainability and routine adoption, this needs to be done without significantly expanding breeding budgets. This requires rethinking how early-stage testing is done in a breeding program. Several approaches have been proposed for incorporating GS in VDPs (Bernardo and Yu, 2007; Cooper et al., 2014; Jacobson et al., 2014; Gaynor et al., 2017; Jarquín et al., 2017; Sukumaran et al., 2018). When evaluating optimal approaches for breeding programs with little or no historical data to train prediction models, strategies that achieve good prediction accuracy from small training sets are critical.

Identifying cost-effective approaches to routinely genotype lines entering the VDP is a critical first step. However, implementation also requires operational capabilities to sample, genotype and generate genomic predictions on a tight turn-around schedule. To do this effectively at scale, advanced breeding informatics systems that include biometrical and quantitative genetics, as well as bioinformatics, are needed. Breeding informatics systems require significant and sustained investment in foundational technologies and computational infrastructure. Fortunately, recent funding initiatives have begun to provide the resources needed to build the foundational capabilities required to modernize and improve the efficiency of public sector breeding programs. The Genomic Open-source Breeding informatics initiative (GOBii)^1^ is one such funding initiative with the goal of building the information systems needed for routine application of genomic technologies to improve efficiency of breeding programs targeting crop improvement in Africa and South Asia. In addition to the project’s focus on genomic technologies, GOBii is also partnering with other open-source breeding informatics initiatives as part of the Excellence in Breeding (EiB)^2^ platform. EiB is being developed as a “complete platform” or set of interconnected tools and strategies designed to increase the efficiency of breeding programs through the adoption of modern technologies and optimal use of breeding resources.

To examine potential approaches for GS implementation, proof-of-concept studies were conducted by the International Crops Research Institute for the Semi-Arid Tropics (ICRISAT) and International Maize and Wheat Improvement Center (CIMMYT) in chickpea and maize, respectively. To examine optimal strategies for routine implementation of GS of lines entering the VDP, three approaches were compared: (i) the development of a dedicated training set (DTS) in parallel to the VDP to serve as a starting point for GS implementation, (ii) splitting full-sib families for training and prediction (FSTS), and (iii) the use of incomplete block designs across environments, or sparse testing (ST), to obtain good prediction accuracies while reducing plot numbers to offset the cost of genotyping. Here we present the results from these studies and discuss strategies for phased implementation of GS in public sector breeding programs. We also highlight breeding informatics capabilities being developed to enable large-scale implementation of genomic breeding strategies.

## Materials and Methods

### Plant Materials

The two datasets from ICRISAT and CIMMYT are described in detail in Roorkiwal et al. (2016, 2018) and Beyene et al. (2019), respectively. Briefly, the chickpea data consists of 315 lines from two distinct chickpea seed-types, Kabuli (*n* = 153) and Desi (*n* = 162), evaluated under rainfed and irrigated regimes in a randomized complete block design with three replicates. All lines were previously genotyped with 2,598 DArT markers (see Roorkiwal et al., 2016 for details). To highlight two contrasting environments, only the rainfed and irrigated environments at ICRISAT from 2013 and 2014 were included in all analyses of the chickpea data.

The maize dataset consists of 849 double haploid (DH) lines from 13 bi-parental families out of the CIMMYT Africa maize breeding program. For demonstration, the three families with the largest family sizes were used for this study (pedigree: CML312/LPS-F64, CML442/LPS-F64, CML536/LPS-F64; size: 91, 108, and 88, respectively). Each DH line was testcrossed to a single tester, and the testcrosses were evaluated in an alpha-lattice incomplete block design with two replications planted in the rainy season with supplemental irrigation as needed in both Kiboko and Kakamega, Kenya, as well as under managed drought conditions during the dry season in Kiboko. The DH lines were genotyped with 9,155 dominant repeat Amplification Sequencing (rAmpSeq) markers at Cornell Life Science Core Laboratory Center, Ithaca, NY, United States (Buckler et al., 2016). Markers were filtered for a minor allele frequency >0.05 and <10% missing values, resulting in 6,785 markers for use in GS.

As the chickpea data consisted of fixed lines generated from many parents, FSTS predictions were not appropriate in this case, and only DTS and ST predictions were compared. In contrast, as the maize dataset consisted of DH generated from three bi-parental crosses, and as such, FSTS and ST were the most appropriate comparisons. In each comparison, the same number of individuals (i.e., half of the individuals for each population) were assigned to the training and test sets.

### Population Structure

Population structure was evaluated using singular value decomposition of the additive genomic relationship matrix, ***K* = UDU’**, where **U** is a matrix of eigenvectors and **D** is a diagonal matrix of eigenvalues. The first two eigenvectors multiplied by their respective eigenvalues were plotted against each other to form a principal component (PC) plot. The proportion of variance explained by each PC is defined as **D**_*ii*_/tr(**D**), where tr() is the trace.

#### Prediction Model

An unstructured univariate genotype by environment interaction model was used to estimate genetic correlations across environments. This can be written as

(2) y=X⁢β+Zu+e

where **y** is the vector of a phenotype in each environment, **X** is the design matrix for the vector β of fixed environmental effects, **Z** is an incidence matrix linking observations in **y** to individuals, **u** is the vector of genetic values and **e** is the vector of residuals. The random effects were both considered centered multivariate normal such that E[**u**] = E[**e**] = 0 and

(3) Var⁢([ue])=[G⊗K00diag⁢(σi2)],

where **G** is the genetic covariance of environments (which must be estimated), and **K** is the additive genomic relationship of individuals, calculated from genetic markers (method I, VanRaden, 2008). Residual variances were considered independent and identically distributed within environment but allowed to differ across environments. Models were fit using the average information algorithm of ASReml (Gilmour et al., 1995; Gilmour, 1997).

Fixed effect values, or Best Linear Unbiased Estimates (BLUEs), were used as true estimated breeding values (EBVs) to compare to the Best Linear Unbiased Predictors (BLUPs), or genomic estimated breeding values (GEBVs). These values were computed using the above model, but allowing **u** to be fixed instead of random, with all observed records included.

### Model Validation

For the Desi and Kabuli comparisons, genomic prediction accuracy was assessed using k-fold cross-validation with 10-fold, where records for a random 10th of the individuals were removed (or masked) from the dataset for each fold. Each fold was predicted before the accuracy was calculated as the Pearson correlation between the all predicted BLUPs and the observed BLUEs. The average accuracy across 10 replicates was used as the estimate of genomic prediction accuracy. This was accomplished among both seed-types, within each seed-type, and across seed-types.

For DTS prediction, the population was randomly split into two sets. Phenotypic records from individuals in one set were removed in both environments before fitting the prediction model with records from the remaining set to predict GEBVs for the missing individuals in both environments. Prediction accuracy of unobserved genotype-environment combinations was then determined using the Pearson correlation of the predicted BLUPs to the observed BLUEs either separately by environment, or by combining predictions across environments. This process was repeated 10 times and averaged to produce an estimate of prediction accuracy.

Similar to DTS prediction, FSTS prediction was accomplished by removing phenotypic records from a random half of the lines within each bi-parental family in all three environments. The remaining individuals were used to fit the prediction model and predict the GEBVs of unobserved individuals in all three environments. Results from ten replicates were averaged to estimate prediction accuracy.

**FIGURE 1:**
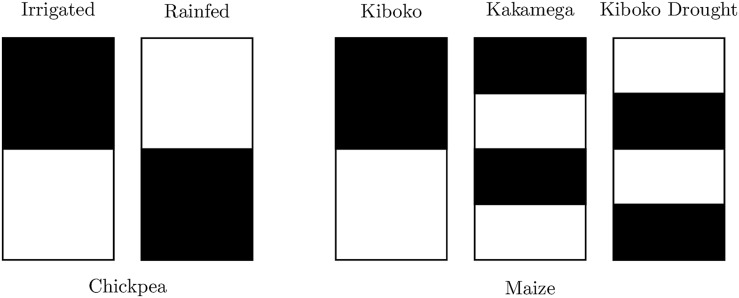
Representation of sparse prediction scheme in maize and chickpea. Black represents records present in the model fit for individuals in each environment, while white represents removed (i.e., missing) records.

Genomic prediction accuracy of ST was determined by again randomly splitting the individuals into two equal sized sets. For ST in chickpea, phenotypic records of one half were removed in the rainfed environment while the records of the other half were removed in the irrigated environment. For ST in the maize dataset, half of the individuals within each family were removed from Kiboko, then further split in half and removed from either Kakamega or Kiboko Drought, along with an additional quarter from the remaining set (see Figure 1). Prediction accuracy of unobserved genotype-environment combinations were then determined using the Pearson correlation to the observed BLUEs either across or within the environment. The mean accuracy and standard deviations of replicates for DTS, FSTS and ST can be found in Supplementary Tables 1, 2.

## Results

**FIGURE 2:**
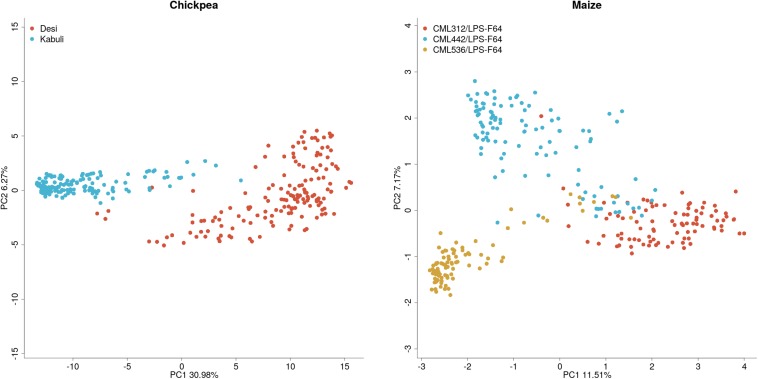
Plots of the first two principal components of the additive genomic relationship matrix for maize and chickpea populations.

Population structures for the chickpea and maize datasets can be found in Figure 2. The principal component plot for the maize dataset shows clustering by population but there is a significant overlap between populations. This is not surprising as the maize dataset consists of half-sibs from multiple populations. In contrast, the chickpea data shows two distinct clusters representing Kabuli and Desi lines, which are both genetically and phenotypically distinct.

To determine whether these two chickpea groups should be combined for training and prediction, cross-validation was conducted among and within each group. Prediction across seed-types was also accomplished to determine if the allele frequency and linkage disequilibrium (LD) pattern is sufficiently shared between seed-types. Results from the cross-validation results are found in Table 1 and Figure 2. High cross-validation accuracies were achieved using the combined dataset, containing both Desi and Kabuli lines in both the training and validation sets, in agreement with Roorkiwal et al. (2016, 2018), however, almost complete loss of predictive ability was observed when one seed-type was used to predict the other (Table 1). Training and validation sets containing only one seed-type were generally less accurate at predicting performance of that seed-type, as compared with training and validation sets containing both seed-types (Table 1).

**TABLE 1:** Genomic prediction accuracies for chickpea GEBVs across environments with various training and test sets estimated using 10-fold cross-validation.

	**All predict all**	**Desi predict Desi**	**Kabuli predict Kabuli**	**Desi predict Kabuli**	**Kabuli predict Desi**
Seed yield	0.48 (0.015)^a^	0.26 (0.029)	0.25 (0.020)	0.08^b^	0.04^c^
Seed weight	0.92 (0.002)	0.76 (0.012)	0.74 (0.014)	0.20	0.58
Biomass	0.50 (0.013)	0.39 (0.019)	0.26 (0.026)	0.11	0.16
Plant height	0.65 (0.011)	0.75 (0.010)	0.42 (0.038)	–0.13	0.16
Days to flower	0.68 (0.007)	0.63 (0.016)	0.56 (0.031)	–0.34	0.07
Days to maturity	0.70 (0.003)	0.53 (0.021)	0.53 (0.038)	–0.16	0.09

**^*a*^Mean prediction accuracy of the Pearson correlation between unobserved BLUPs and observed BLUEs. Standard deviation of ten replicates is shown in parentheses. ^*b*^All Desi lines used to predict all Kabuli lines (no cross-validation). ^*c*^All Kabuli lines used to predict all Desi lines (no cross-validation).**

**FIGURE 3:**
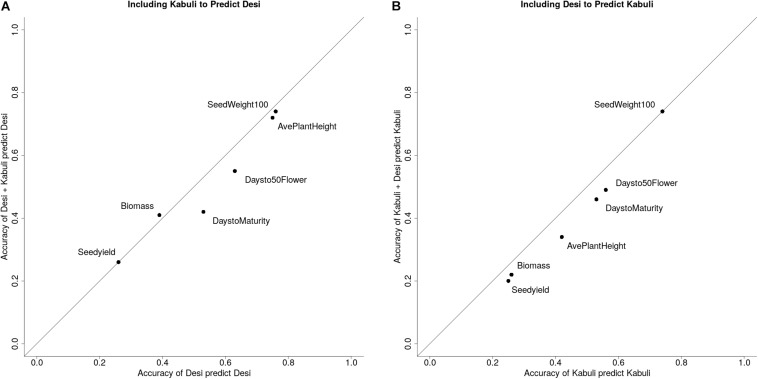
Prediction accuracies for Desi and Kabuli with different structures for training and validation sets. **(A)** Prediction accuracy of using only Kabuli lines to predict Kabuli lines vs. using both Desi and Kabuli lines to predict Kabuli lines. **(B)** Prediction accuracy of using only Desi lines to predict Desi lines vs. using both Kabuli and Desi lines to predict Desi lines.

To determine whether the high prediction accuracies seen using Desi and Kabuli in both training and validation sets were due to the prediction of group differences between Desi and Kabuli, or due to predictions of phenotype variation within seed-type, we then compared (1) single seed-type training sets to predict phenotypes for the same seed-type, with (2) both seed-types to predict phenotypes for a single seed-type. Higher prediction accuracies were generally observed when the training population was consisted of a single seed-type (Figure 3).

**FIGURE 4:**
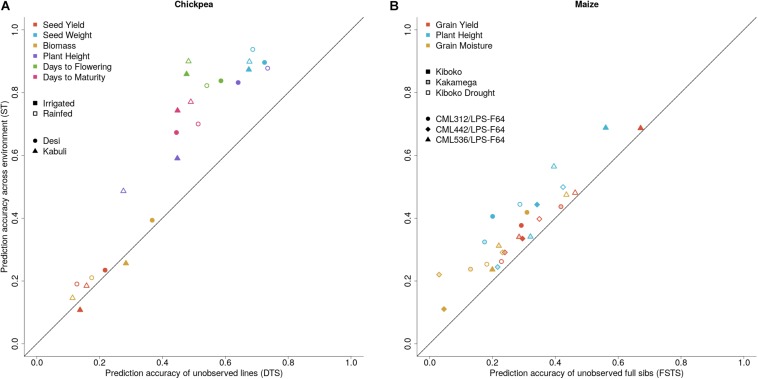
Prediction accuracies for sparse testing (ST) vs. **(A)** dedicated training set (DTS) prediction accuracies in chickpea lines across six traits and two water regimes, and **(B)** Full-sib prediction accuracies (FSTS) in maize across three traits and three environments.

Genetic correlations between environments vary across traits and range from moderate to high (Table 2). Results from cross-validation comparing sparse testing to prediction using historical information can be found in Figure 4. Results show that across traits, the sparse testing approach consistently achieves prediction accuracies that are as good or higher, which agrees with similar studies in wheat (Jarquín et al., 2017; Sukumaran et al., 2018). Unsurprisingly, the relative improvement in performance increases for traits with higher genetic correlations across environments.

**TABLE 2:** Plot level heritabilities and genetic correlations across rainfed and irrigated environments for chickpea.

**Chickpea**		**Desi**		**Kabuli**	
		**Rainfed**	**Irrigated**	**Rainfed**	**Irrigated**
Seed yield	Rainfed	0.38^a^	0.24^b^	0.29	0.1
	Irrigated		0.32		0.16
Seed weight	Rainfed	0.59	0.88	0.65	0.83
	Irrigated		0.76		0.74
Biomass	Rainfed	0.21	0.41	0.27	0.25
	Irrigated		0.28		0.11
Plant height	Rainfed	0.54	0.87	0.42	0.73
	Irrigated		0.64		0.49
Days to flowering	Rainfed	0.51	0.91	0.55	0.97
	Irrigated		0.6		0.67
Days to maturity	Rainfed	0.36	0.82	0.49	0.89
	Irrigated		0.34		0.38

**TABLE 3:** Plot level heritabilities and genetic correlations across three environments for maize.

**Maize**				

		**Kiboko**	**Kakamega**	**Kiboko drought**
Yield	Kiboko	0.30	0.54	0.72
	Kakamega		0.25	0.40
	Kiboko drought			0.30
Moisture	Kiboko	0.05	0.55	0.98
	Kakamega		0.45	0.31
	Kiboko drought			0.19
Plant Height	Kiboko	0.36	0.86	0.97
	Kakamega		0.27	0.77
	Kiboko drought			0.32

**^*a*^Plot level heritabilities within each environment are represented on the diagonal. ^*b*^The above diagonal is the estimated genetic correlation of environments.**

## Discussion

### Strategy for Phased Implementation

**FIGURE 5:**
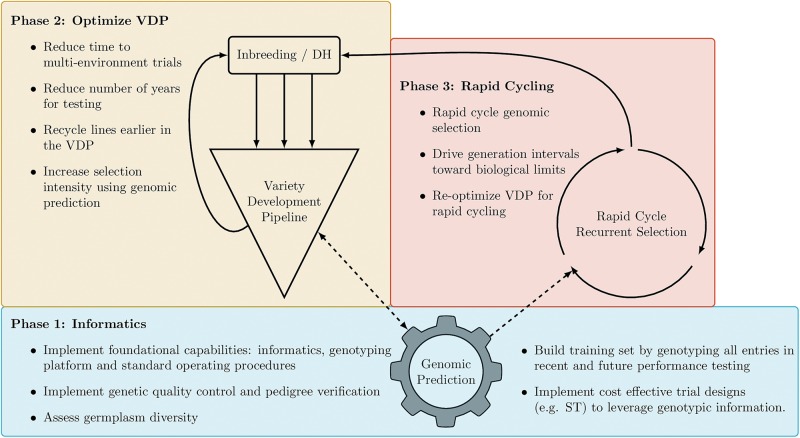
Recommended implementation of genomic information into a breeding program. Phase 1 **(blue)**, Phase 2 **(yellow)**, and Phase 3 **(red)**. Solid lines indicate the flow of genetic materials, while dashed lines indicate the flow of information.

Routine implementation of genomic information represents significant changes in the way plant breeding programs operate and how breeding decisions are made. To facilitate routine implementation, we recommend a phased implementation strategy (Figure 5). In Phase 1 the goal should be to establish informatics capabilities to successfully implement GS and optimize trial designs, such as ST and FSTS, to build appropriate training datasets in a cost-effective manner. While the focus of this paper is the routine implementation of GS, it should be noted that routine genotyping of all entries in the VDP will immediately enable genetic quality control and pedigree verification which can improve the overall efficiency of the breeding pipeline by identifying errors early in the screening process. Once the accuracies of genomic prediction models are validated in a breeding program, Phase 2 of implementation should focus on increasing selection intensity in the early stages of the VDP, reducing the number of seasons in which varieties are tested prior to release and recycling lines as new parents earlier in the testing process. Finally, Phase 3 would focus on the implementation of rapid-cycle recurrent selection to reduce generation intervals towards the biological limits of the species. The proposed phases account for the dependencies and logistical complexities of implementation, as well as the size of the training set needed to maintain accurate predictions. Phase 1 assumes a breeding program is starting with very little combined genotypic and phenotypic data to train prediction models. Given that most public sector breeding programs in Africa and South Asia have yet to initiate routine genotyping of lines entering the VDP, the key first step is to implement capabilities and cost-effective strategies to routinely genotype lines entering early-stage testing and generate accurate predictions with limited training data. This is a key focus of this study and of projects like GOBii and the High Throughput Genotyping (HTPG) project, both funded by the Bill and Melinda Gates foundation, which are working to increase genetic gains and improve the efficiency of breeding programs serving Africa and South Asia.

The data used in this study were collected on crops with different breeding approaches and different initial strategies for building training sets and applying GS in early-stage trials. These contrasting crops make for an interesting dataset for testing widely applicable strategies for the initial adoption of GS approaches. The chickpea training set was developed to represent the full diversity of ICRISAT chickpea breeding programs, both Kabuli and Desi, for the purpose of predicting the performance of new lines prior to preliminary yield trials. The combined chickpea training set may be good at distinguishing phenotypic differences between the two known groups, but less accurate at discriminating within groups. The inability to predict across seed-types demonstrates that population specific allele frequency and LD patterns appear to be driving the observed prediction accuracy. While using Kabuli lines to predict the performance of Desi, and vice versa, may be viewed as an extreme case, the large decreases in prediction accuracy when compared to the use of Desi to predict Desi and Kabuli to predict Kabuli highlight the importance of building appropriate training sets.

In the chickpea case, the estimates of prediction accuracy using both seed-types were overly optimistic and could have disappointed and discouraged funders of these early GS efforts. Indeed, many reported genomic prediction accuracies are likely upward biased when it comes to selection, as the (unobserved) accuracy of new lines formed from relatively few crosses will not be inflated by the same degree of population structure within the diverse training population. The inability to predict across demonstrates that the two seed-types comprise effectively separate breeding programs and should be treated as such for training population designs in order to provide realistic expectations to funders. It may be prudent to refrain from reporting accuracies in diverse populations, instead focusing on the average of within group/family to guide expectations.

It has been shown that, when assuming the infinitesimal model, the expected prediction accuracy is a function of population structure, trait heritability, training set size, and the accuracy with which genomic relationships calculated using genetic markers estimate the true genomic relationships at the QTL regions controlling the trait of interest (Goddard, 2009; Daetwyler et al., 2010; Goddard et al., 2011). The latter is a function of both marker density and the number of independent chromosomal segments segregating between the training set and the target set of lines for prediction. Strategies that utilize training sets containing lines closely related to the target lines for prediction reduces the number of independently segregating chromosomal segments, which in turn increases prediction accuracy. When LD is high, as it is within close relatives, small training sets and mid to low-density marker platforms can adequately capture the genetic information required for prediction (Schopp et al., 2017; Brauner et al., 2019). A straight-forward approach to ensuring training data is closely related to new lines being developed in the breeding programs is to adopt a dual purpose line development and VDP approach to building training sets, where the VDP serves the additional purpose of providing training data to continuously update predictive models (Schopp et al., 2017; Brauner et al., 2019). While this certainly isn’t a groundbreaking revelation, it does provide a clear target for the initial step in implementing GS in a breeding program: cost-effective genotyping of all lines entering the VDP. The general concept of maintaining reasonably close genetic relationships between the germplasm in the line development program and germplasm in the VDP is an important consideration when balancing the effectiveness of the long-term GS implementation strategy with the need to diversify the germplasm base. Maintaining diversity is important for sustained long-term genetic gain as well as response to evolving breeding targets. While GS shows substantial promise for improving breeding program efficiency, it requires a thoughtful germplasm strategy to maximize long-term effectiveness.

When comparing approaches to initiate a dual purpose VDP, the ST approach consistently outperforms both FSTS and DTS in terms of prediction accuracy. Given the differences in crops and population structure of the training sets in this study, the fact that ST delivered higher prediction accuracies in both cases indicates that it could be a robust strategy across crops and breeding programs. It should be noted that the ST method does necessitate the generation of seed from all lines, where the FSTS does not, however, the amount of seed required is less, presenting the potential for time savings during seed multiplication for inbred crops. In lower throughput programs where seed multiplication occurs in the field, this could allow material to enter the VDP an entire year earlier. However, for hybrid crops, the cost implication of seed multiplication for ST is greater since hybrid crops require testcrossing all candidates. The tradeoffs between cost and accuracy need to be carefully considered when considering implementation strategies.

Traits that benefited most from the sparse testing approach were of moderate to high heritability. Traits with low heritability, such as seed yield, also tended to have low genetic correlations across environments. Often, moderate to high heritability traits are under selection in small plot trials during seed multiplication, meaning sparse testing may not be as advantageous for these cases as indicated here. More importantly, the observation of all lines in the field, as is done in ST, allows for a breeder to identify and cull lines with other undesirable, but highly heritable traits, before they enter into extensive field trials. Sparse testing also presents opportunities for cross program collaboration, including across countries or continents. If both programs share a marker platform, implementation of germplasm sharing could be expedited by predicting performance in the other program, and exchange of promising materials for the other environment(s). However, this may be limited to programs that already share related materials which can be reliably predicted.

While prediction accuracy is a major factor in determining the best approaches to implement GS, the cost and complexity of implementation must also be considered. For simplicity and ease of comparison, the same number of plots were used in training predictive models for each approach presented here. This does not mean that each approach would have roughly the same cost or the same efficiency in VDP design. The FSTS approach has the advantage of reducing the number of lines for which seed must be produced for yield trial testing as with this approach phenotypic data is not collected on all genotyped lines. The DTS approach enables prediction of new lines prior to the collection of any information on the line itself or on full-sibs, but requires significant initial investment to develop the training set. Thus, it is difficult to envision an implementation that is cost neutral in terms of the total breeding budget. The ST approach combines genomic prediction and advancement decisions into a single analysis. The fact that implementation can be viewed as a change in experimental design is appealing, but it does increase the complexity of models that need to be run to advance lines through the VDP. In an ST approach, genomic relationship matrices need to be calculated for variety trials and used in mixed models for variety advancements. This adds complexity to the traditional advancement process that could quickly overwhelm even a moderate sized breeding program without breeding informatics tools to support the process.

It is important to note that incomplete block designs typically have some explicit genetic overlap, with some lines shared across each pair of environments, as is the case here in maize. With this overlap, the genetic correlation of environments can be estimated even when lines are considered independent. When genotypes are available, however, the genetic correlation of environments can be estimated without the need to replicate any lines across environments. These correlations are instead estimated through replication of alleles across environments, as is the case here in chickpea. The ST approach does require estimation of genetic correlations across environments, and this should be taken into account when designing multi-environment trials. Generally, greater levels of genetic overlap will increase the precision of these correlation estimates.

While these results provide some general guidance on promising approaches for initial implementation of routine GS, the optimal implementation strategy will depend on the specifics of each breeding pipeline. The heritability of traits of interest, cost of phenotyping, amount of historical data available for training prediction models, field testing resources, structure of breeding populations, and access to cost-effective genotyping platforms are all factors that will influence decisions about optimal approaches for implementation of GS. Even within a program there may be a need for hybrid approaches given the expected prediction accuracy for a given population using historical data. It is recommended that any breeding program test the potential efficiency of new approaches using simulation prior to implementation. Fortunately, there are freely available simulation packages (Faux et al., 2016; Yabe et al., 2017), and EiB is working directly with public breeding programs in Africa and South Asia to conduct simulations and make recommendations for optimal breeding pipeline designs.

### Breeding Informatics

Implementation of any of the approaches examined in this study will require full integration of genomic information into routine breeding decisions, requiring a shift in how data is viewed and handled in a breeding program. The need to build a large training set through a dual purpose VDP means that variety testing trials can no longer be viewed as independent experiments for the identification and advancement of superior varieties. The data collected should be treated as a resource for increasing understanding of breeding germplasm and improving the accuracy of breeding decisions (Spindel and McCouch, 2016). The capability to combine genotypic data with phenotypic data collected across experiments, environments, and seasons will be critical for success. While challenging in and of itself, the narrow timelines between harvesting yield trials and planting nurseries to generate seed for the next season make it infeasible to implement these approaches without effective data management and analytic platforms. To bring genomic information into routine breeding decisions and enable access to valuable data resources, information systems are required to track samples, store genomic and phenotypic information, and implement analysis pipelines to merge data from multiple sources and conduct advanced analytics to guide decision making on tight schedules. In addition, a standardized, low-cost and robust genotyping platform with short turn-around time is essential to delivering high-quality genotyping data in a timely fashion.

**FIGURE 6:**
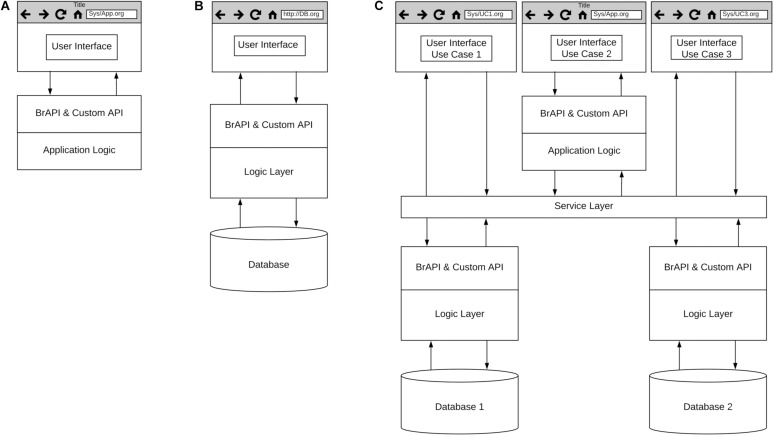
High level architecture for breeding software. **(A)** Applications, **(B)** Databases, and **(C)** Breeding management systems.

To address this critical need, GOBii, EiB, and several other projects are working with public sector breeding programs to build and deploy the foundational capabilities needed to digitize breeding data, support breeding processes and implement GS routinely. Given the size and capacity of many public sector breeding programs, open-source breeding software needs to be both scalable and customizable to meet the needs of diverse crop breeding programs. To accomplish this communities of practice associated with projects like the Breeding Application Program Interface (BrAPI; Selby et al., 2019) and EiB are working to develop best practices and standards to enable interoperability of software being developed across multiple development teams and projects. Figure 6 represents a high-level, generic architecture focused on the development of web-based breeding software tools. The use of web-based tools enables cloud deployment of complex systems and software as a service (SaaS) for scalability. Common standards for Application Program Interfaces (API), such as BrAPI, will enable customization for a variety of breeding processes. It should be noted that these development efforts are not limited to web-based applications as it is recognized that certain breeding activities will need to be conducted offline.

Several development teams are building applications designed to support specific breeding processes (Figure 6A), and several examples of these applications can be found on the BrAPI website^3^. Projects like GOBii are focused on building databases designed to achieve optimal performance with specific data types (Figure 6B). The GOBii genomic data management system (GOBii GDM) is designed to store multiple genomic data types and is built on technology that enables fast querying of large genomic datasets (Nti-Addae et al., 2019). The GOBii system utilizes RESTful APIs and the BrAPI standard to enable connections to breeding management systems and breeding analytics pipelines being developed by EiB and other open-source software development projects (Tecle et al., 2014; Ribaut and Ragot, 2019). Finally, several projects such as the EiB Enterprise Breeding System (EBS) and the USDA ARS Breeding Insight^4^ are developing systems composed of multiple applications and databases for end-to-end support of breeding processes, leveraging existing breeding software and databases when feasible (Figure 6C).

To enable cost-effective genotyping, EiB, in collaboration with the High Throughput Genotyping (HTPG)^5^ project, are sourcing genotyping platforms to reduce the cost of mid-density genotyping (1,000–2,000 markers) to a price per line that is comparable to the cost of running a single yield trial plot. Using the HTPG platform, EiB is implementing low-cost genotyping services for public sector breeding programs. Access to these low-cost genotyping services, combined with open-source databases and analytic pipelines greatly reduces barriers to cost-effective implementation of GS strategies and should pave the way for routine use of GS in public sector breeding programs in the near future.

Notably, adoption of new technology demands a skilled workforce. Rapid creation, quality control and turnover of genotypic and phenotypic data will be necessary to make and implement breeding decisions. This will result in many moving parts, and all these steps require a high degree of skill. Many programs will need to adopt a team-oriented approach where expertise is split across many individuals, with enough overlap for effective communication. Future members of plant breeding teams will need skills and expertise outside of what has traditionally been associated with plant breeding. Expertise in database management, machine learning, biometrics, software development, engineering, and operations research will be needed to augment the biology, genetics, and agronomy skills of the team. We acknowledge that building this expertise for every program would be impractical, therefore movement towards regional networks with shared services and expertise will be necessary.

## Conclusion

There are several barriers to routine implementation of GS at a breeding program scale. These barriers are currently being addressed and we foresee movement towards routine adoption in several public breeding programs. We suggest that breeding programs approach the implementation of GS in a phased approach with the initial step being the routine genotyping of all materials that are evaluated for yield. These materials will be genetically and environmentally close to the materials to be predicted in later stages. We stress that genotyping should be a regular process instead of a series of isolated efforts as is often practiced today. Modification of a traditional variety development pipeline will include implementation of experimental designs that optimize resources allocated to phenotyping and genotyping. Changes in experimental designs and VDP structure should focus on reductions in replications, sparse testing, and faster germplasm turnover. Marker data must be seamlessly integrated with pedigree information, phenotypes, and experimental design to facilitate data processing and analysis for making breeding decisions at a fast turnover rate. Adoption of standardized databases and analysis platforms is necessary to streamline decision making processes. Many of these platforms exist or are currently being constructed, but adoption will be key to successful implementation of GS into the 21st century public breeding program.

## Data Availability Statement

The datasets analyzed in this article are not publicly available. Requests to access the datasets should be directed to Yoseph Beyene, y.beyene@cgiar.org (maize); Rajeev Varshney, r.k.varshney@cgiar.org (chickpea).

## Author Contributions

NS analyzed the chickpea data, contributed to the design of the study, and drafting of the manuscript. SA analyzed the maize data, contributed to the design of the study, and the drafting of the manuscript. KR, MO, and EJ contributed to the design of the study and drafting of the manuscript. YB, RV, MG, PG, XZ, CB, and MR contributed to the design of the study and collection of phenotypic and genotypic data. CH, AR, KD, JC, and PP-R contributed to the curation, QC, and analysis of the data. SG and SM contributed to the drafting of the manuscript.

## Conflict of Interest

The authors declare that the research was conducted in the absence of any commercial or financial relationships that could be construed as a potential conflict of interest.

## Footnotes

1 www.gobiiproject.org

2 www.excellenceinbreeding.org

3 https://brapi.org/brapps

4 https://www.breedinginsight.org/about

5 cegsb.icrisat.org/high-throughput-genotyping-project-htpg/
