# Supplementary material for: Strategies for Effective Use of Genomic Information in Crop Breeding Programs Serving Africa and South Asia
Source: Front Plant Sci. 2020 Mar 27;11:353. doi: 10.3389/fpls.2020.00353 (PMC7119190; doi:10.3389/fpls.2020.00353)
Supplement: Supplementary file 1 [file Table_1.DOCX]

**Supplementary Table 1.** Mean prediction accuracy and standard deviation of DTS and ST methods for chickpea by environment and seed type.

| Environment | Trait | Seed Type | DTS | ST |
| --- | --- | --- | --- | --- |
| Irrigated | Plant Height | Desi | 0.64 (0.053) | 0.83 (0.035) |
| Irrigated | Biomass | Desi | 0.37 (0.081) | 0.39 (0.089) |
| Irrigated | Days to Flowering | Desi | 0.59 (0.046) | 0.84 (0.024) |
| Irrigated | Days to Maturity | Desi | 0.44 (0.08) | 0.67 (0.054) |
| Irrigated | Seed Weight | Desi | 0.72 (0.051) | 0.90 (0.021) |
| Irrigated | Seed Yield | Desi | 0.22 (0.092) | 0.23 (0.093) |
| Rainfed | Plant Height | Desi | 0.73 (0.040) | 0.88 (0.024) |
| Rainfed | Biomass | Desi | 0.17 (0.124) | 0.21 (0.114) |
| Rainfed | Days to Flowering | Desi | 0.54 (0.055) | 0.82 (0.047) |
| Rainfed | Days to Maturity | Desi | 0.51 (0.058) | 0.70 (0.034) |
| Rainfed | Seed Weight | Desi | 0.69 (0.055) | 0.94 (0.020) |
| Rainfed | Seed Yield | Desi | 0.13 (0.117) | 0.19 (0.159) |
| Irrigated | Plant Height | Kabuli | 0.45 (0.064) | 0.59 (0.070) |
| Irrigated | Biomass | Kabuli | 0.28 (0.091) | 0.26 (0.095) |
| Irrigated | Days to Flowering | Kabuli | 0.48 (0.075) | 0.86 (0.036) |
| Irrigated | Days to Maturity | Kabuli | 0.45 (0.076) | 0.74 (0.057) |
| Irrigated | Seed Weight | Kabuli | 0.67 (0.035) | 0.87 (0.019) |
| Irrigated | Seed Yield | Kabuli | 0.14 (0.094) | 0.11 (0.105) |
| Rainfed | Plant Height | Kabuli | 0.28 (0.105) | 0.49 (0.133) |
| Rainfed | Biomass | Kabuli | 0.11 (0.088) | 0.15 (0.075) |
| Rainfed | Days to Flowering | Kabuli | 0.48 (0.067) | 0.90 (0.028) |
| Rainfed | Days to Maturity | Kabuli | 0.49 (0.078) | 0.77 (0.057) |
| Rainfed | Seed Weight | Kabuli | 0.68 (0.032) | 0.90 (0.013) |
| Rainfed | Seed Yield | Kabuli | 0.16 (0.110) | 0.18 (0.109) |

**Supplementary Table 2.** Mean prediction accuracy and standard deviation of FSTS and ST methods for maize by environment and family.

| Environment | Trait | Pedigree | FSTS | ST |
| --- | --- | --- | --- | --- |
| Kiboko Optimal | Grain Yield | CML312/LPS-F64 | 0.29 (0.12) | 0.38 (0.08) |
| Kiboko Optimal | Plant Height | CML312/LPS-F64 | 0.20 (0.11) | 0.41 (0.10) |
| Kiboko Optimal | Grain Moisture | CML312/LPS-F64 | 0.31 (0.13) | 0.42 (0.11) |
| Kakamega | Grain Yield | CML312/LPS-F64 | 0.42 (0.08) | 0.44 (0.10) |
| Kakamega | Plant Height | CML312/LPS-F64 | 0.18 (0.08) | 0.32 (0.10) |
| Kakamega | Grain Moisture | CML312/LPS-F64 | 0.13 (0.10) | 0.24 (0.09) |
| Kiboko Drought | Grain Yield | CML312/LPS-F64 | 0.23 (0.09) | 0.26 (0.08) |
| Kiboko Drought | Plant Height | CML312/LPS-F64 | 0.29 (0.12) | 0.44 (0.11) |
| Kiboko Drought | Grain Moisture | CML312/LPS-F64 | 0.18 (0.05) | 0.25 (0.07) |
| Kiboko Optimal | Grain Yield | CML442/LPS-F64 | 0.29 (0.11) | 0.33 (0.01) |
| Kiboko Optimal | Plant Height | CML442/LPS-F64 | 0.34 (0.11) | 0.44 (0.06) |
| Kiboko Optimal | Grain Moisture | CML442/LPS-F64 | 0.05 (0.12) | 0.11 (0.15) |
| Kakamega | Grain Yield | CML442/LPS-F64 | 0.24 (0.05) | 0.29 (0.07) |
| Kakamega | Plant Height | CML442/LPS-F64 | 0.22 (0.07) | 0.24 (0.05) |
| Kakamega | Grain Moisture | CML442/LPS-F64 | 0.03 (0.15) | 0.22 (0.08) |
| Kiboko Drought | Grain Yield | CML442/LPS-F64 | 0.30 (0.07) | 0.37 (0.1) |
| Kiboko Drought | Plant Height | CML442/LPS-F64 | 0.42 (0.10) | 0.50 (0.10) |
| Kiboko Drought | Grain Moisture | CML442/LPS-F64 | 0.23 (0.02) | 0.2 (0.05) |
| Kiboko Optimal | Grain Yield | CML536/LPS-F64 | 0.69 (0.08) | 0.69 (0.08) |
| Kiboko Optimal | Plant Height | CML536/LPS-F64 | 0.69 (0.07) | 0.69 (0.07) |
| Kiboko Optimal | Grain Moisture | CML536/LPS-F64 | 0.24 (0.09) | 0.24 (0.09) |
| Kakamega | Grain Yield | CML536/LPS-F64 | 0.28 (0.09) | 0.34 (0.07) |
| Kakamega | Plant Height | CML536/LPS-F64 | 0.32 (0.15) | 0.34 (0.10) |
| Kakamega | Grain Moisture | CML536/LPS-F64 | 0.22 (0.10) | 0.31 (0.05) |
| Kiboko Drought | Grain Yield | CML536/LPS-F64 | 0.30 (0.07) | 0.48 (0.07) |
| Kiboko Drought | Plant Height | CML536/LPS-F64 | 0.42 (0.10) | 0.56 (0.16) |
| Kiboko Drought | Grain Moisture | CML536/LPS-F64 | 0.23 (0.02) | 0.47 (0.04) |
